# Proviral Turnover During Untreated HIV Infection Is Dynamic and Variable Between Hosts, Impacting Reservoir Composition on ART

**DOI:** 10.3389/fmicb.2021.719153

**Published:** 2021-08-19

**Authors:** Kelsie Brooks, F. Harrison Omondi, Richard H. Liang, Hanwei Sudderuddin, Bradley R. Jones, Jeffrey B. Joy, Chanson J. Brumme, Eric Hunter, Zabrina L. Brumme

**Affiliations:** ^1^Emory Vaccine Center, Emory University, Atlanta, GA, United States; ^2^Faculty of Health Sciences, Simon Fraser University, Burnaby, BC, Canada; ^3^British Columbia Centre for Excellence in HIV/AIDS, Vancouver, BC, Canada; ^4^Bioinformatics Program, University of British Columbia, Vancouver, BC, Canada; ^5^Department of Medicine, University of British Columbia, Vancouver, BC, Canada; ^6^Department of Pathology and Laboratory Medicine, Emory University School of Medicine, Atlanta, GA, United States

**Keywords:** HIV, persistence, reservoir, within-host phylogenetic analysis, proviral half-life

## Abstract

Human immunodeficiency virus (HIV) can persist as an integrated provirus, in a transcriptionally repressed state, within infected cells. This small yet enduring pool of cellular reservoirs that harbor replication-competent HIV is the main barrier to cure. Entry of viral sequences into cellular reservoirs begins shortly after infection, and cells containing integrated proviral DNA are extremely stable once suppressive antiretroviral therapy (ART) is initiated. During untreated HIV infection however, reservoir turnover is likely to be more dynamic. Understanding these dynamics is important because the longevity of the persisting proviral pool during untreated infection dictates reservoir composition at ART initiation. If the persisting proviral pool turns over slowly pre-ART, then HIV sequences seeded into it during early infection would have a high likelihood of persisting for long periods. However, if pre-ART turnover was rapid, the persisting proviral pool would rapidly shift toward recently circulating HIV sequences. One-way to estimate this turnover rate is from the age distributions of proviruses sampled shortly after therapy initiation: this is because, at the time of sampling, the majority of proviral turnover would have already occurred prior to ART. Recently, methods to estimate a provirus’ age from its sequence have made this possible. Using data from 12 individuals with HIV subtype C for whom proviral ages had been determined phylogenetically, we estimated that the average proviral half-life during untreated infection was 0.78 (range 0.45–2.38) years, which is >15 times faster than that of proviral DNA during suppressive ART. We further show that proviral turnover during untreated infection correlates with both viral setpoint and rate of CD4+ T-cell decline during this period. Overall, our results support dynamic proviral turnover pre-ART in most individuals, which helps explain why many individuals’ reservoirs are skewed toward younger HIV sequences. Broadly, our findings are consistent with the notion that active viral replication creates an environment less favorable to proviral persistence, while viral suppression creates conditions more favorable to persistence, where ART stabilizes the proviral pool by dramatically slowing its rate of decay. Strategies to inhibit this stabilizing effect and/or to enhance reservoir turnover during ART could represent additional strategies to reduce the HIV reservoir.

## Introduction

Viruses evade host immune detection through various strategies, and among these is the ability to persist in a transcriptionally repressed state within host cells (Simmons et al., 2013). Human immunodeficiency virus (HIV) is no exception. Like all retroviruses, HIV integrates its genome into that of its host cell, and a small number of cells harboring integrated proviruses persist long-term *in vivo*, even during suppressive antiretroviral therapy (ART). While most persisting proviruses harbor genetic defects (Ho et al., 2013; Bruner et al., 2016; Imamichi et al., 2016), a minority are genomically intact and have the potential to produce infectious HIV at any time. These cellular reservoirs are the major barrier to achieving ART-free HIV remission or cure, and would need to be reduced, inactivated or eliminated to achieve these goals.

It is clear that cellular reservoirs are established very early in infection (Ananworanich et al., 2012, 2015; Hocqueloux et al., 2013) and that they are extremely stable during long-term ART (Siliciano et al., 2003; Crooks et al., 2015; Golob et al., 2018; Peluso et al., 2020). Pre-ART proviral dynamics however are less well understood, though recent data suggest that proviral turnover is more rapid during untreated infection than during suppressive therapy (Brodin et al., 2016; Abrahams et al., 2019; Pankau et al., 2020). As pre-ART proviral longevity determines reservoir composition at ART initiation, it is critical for us to understand these dynamics if we are to develop approaches to inactivate or eliminate HIV reservoirs.

Recent studies interpreting on-ART proviral diversity in context of HIV’s within-host evolutionary history have revealed that the persisting proviral pool is typically enriched in HIV variants that circulated during later chronic infection, though it is not uncommon to recover proviruses that are identical or similar to the transmitted/founder virus or its direct descendants (Brodin et al., 2016; Jones et al., 2018; Brooks et al., 2020; Pankau et al., 2020). Though these analyses captured total (i.e., both defective and intact) proviruses, a recent study that recovered replication-competent proviruses on ART revealed that, while some dated back to acute/early infection, the majority represented sequences that circulated in the year before ART initiation (Abrahams et al., 2019). Observing acute/early infection sequences at generally low frequencies in both the persisting proviral pool and the replication-competent reservoir, but nevertheless to varying extents between individuals, suggests that proviral turnover pre-ART is dynamic, and that rates of turnover vary between individuals (Brodin et al., 2016; Abrahams et al., 2019; Pankau et al., 2020). Two recent studies have inferred these rates from the estimated ages of proviruses sampled on ART, yielding estimated pre-ART proviral half-lives of 9 months (Brodin et al., 2016) and 25 months (Pankau et al., 2020), though in the latter study, estimates ranged from 7 to 68 months depending on the individual and the HIV gene analyzed. Though variable, these average rates are nevertheless much shorter than both the half-life of proviral DNA on suppressive ART, which is estimated as >10 years (Golob et al., 2018; Peluso et al., 2020), and the half-life of the replication-competent reservoir on ART, which is estimated as 44 months or longer (Siliciano et al., 2003; Gandhi et al., 2020; Peluso et al., 2020). Together, these observations are consistent with more rapid proviral turnover during untreated compared to treated infection.

In total however, existing pre-ART proviral half-life estimates are based on data from only 16 individuals: a group of 10 persons with predominantly HIV subtype B infections and 6 women with super-infection (Brodin et al., 2016; Pankau et al., 2020). No studies to our knowledge have calculated pre-ART proviral turnover rates in a gender-balanced group of individuals with HIV subtype C, the most prevalent subtype globally, using such approaches. Furthermore, the correlates of within-host pre-ART proviral decay rate remain undefined. To address these gaps, we studied a Zambian cohort of 12 individuals (seven men and five women) with HIV subtype C infections for whom the integration dates of proviruses sampled on ART had been determined phylogenetically (Brooks et al., 2020), and leveraged these data to infer within-host proviral decay rates during untreated HIV infection.

## Materials and Methods

### Participants and Ethics Statement

This study leveraged 12 longitudinal HIV sequence datasets that were originally used to characterize on-ART proviral diversity and age distributions among participants of a Zambian seroconverters cohort (Brooks et al., 2020). The original study featured 13 participants; here we analyze 12, all of whom had single-variant HIV subtype C (one individual with mixed infection was excluded). As described in the original publication, plasma HIV envelope (*env*) sequences were sampled at seroconversion and two additional time points prior to ART initiation, and proviruses were sampled at least once during suppressive ART (for four participants, proviruses were sampled twice). All HIV sequences were characterized using single-genome amplification followed by next-generation sequencing on the Pacific Biosciences SMRTbell platform. The GenBank Accession numbers of studied sequences were MT194125 – MT194771 and MT194898 – MT195535. All participants gave written informed consent. The cohort study was approved by the University Teaching Hospital Ethics Committee in Lusaka, Zambia, and additional approvals for sample and data use were granted by the Institutional Review Boards of Emory University, Simon Fraser University, and Providence Health Care/University of British Columbia.

### Proviral Age Estimation Using a Within-Host Phylogenetic Approach

Proviral age estimation was performed phylogenetically as previously described (Brooks et al., 2020). Briefly, defective HIV sequences harboring hypermutation, ambiguous bases, or evidence of within-host recombination within *env* were removed from each participant’s dataset. Each participant’s remaining plasma HIV RNA sequences collected between seroconversion and ART initiation, along with proviral sequences collected on ART, were used to infer a maximum likelihood phylogeny. The phylogeny was then rooted at the location that maximized the correlation between the root-to-tip distances and collection dates of the pre-ART plasma HIV RNA sequences, where this root represented the inferred transmitted/founder virus. A linear regression relating the pre-ART HIV RNA root-to-tip distances to their sampling times was then fit, where the slope of this line represented the host-specific rate of HIV evolution pre-ART. The regression was then used to convert the root-to-tip distance of each proviral sequence to its “creation” (i.e., integration) date, with associated 95% confidence intervals (CI). As described in the original paper, all 12 datasets yielded strong molecular clock signal (Brooks et al., 2020), allowing us to infer the integration dates of 259 intact proviruses, 253 of which were distinct [median 20, interquartile range (IQR) 15–25 distinct intact proviruses per participant]. Each participant’s proviruses were then grouped into “bins” by their estimated year of integration (one “bin” per year preceding ART initiation), though a sensitivity analysis was also undertaken where the point estimates were used directly without binning. In the primary analysis, integration date estimation was only performed on the 253 distinct proviral sequences under the assumption that “replicate” sequences arose through clonal expansion and not through individual integration events. Nevertheless, sensitivity analyses that incorporated all intact proviruses, including the six “replicate” sequences (observed in only four participants), were also performed. For simplicity, proviruses whose integration date point estimate fell after the ART initiation date (all of which had 95% CIs that extended to before ART) were assigned to the ART initiation date.

### Illustrating How Pre-ART Proviral Half-Lives Shape Proviral Composition Using a Dynamical Mathematical Model of Reservoir Seeding and Decay

To illustrate how proviral clearance rates during untreated infection shape proviral composition at ART initiation, we implemented a published dynamical mathematical model of HIV infection (Pankau et al., 2020). This model comprises a set of ordinary differential equations that describe within-host cell and virus concentrations over time, where these equations include susceptible target cells, actively and latently infected cells that can produce viable virus, actively and latently infected cells that *cannot* produce viable virus, the virus itself, and an immune response. The model assumes that HIV sequences enter the latent pool at a rate proportional to their abundance in plasma at the time, an assumption that is supported by the observation that latently infected cell frequency correlates positively with the area under the pretreatment viral load curve (Archin et al., 2012). In a subsequent independent step, proviruses then decay out of the latent pool at a constant, exponential rate to produce predictions of what the age distribution of the proviral pool would be if sampled on ART, given different rates of pre-ART decay. We reproduce the model here for clarity.

Let *S* represent the susceptible compartment. Let *A*_*P*_ and *L*_*P*_ represent the compartments of actively and latently infected cells that are productively infected and produce viable virus; likewise let *A*_*U*_ and *L*_*U*_ represent the compartments of actively and latently infected cells that are *unproductively* infected and cannot produce viable virus. Let *V* represent viremia and let *E* represent the adaptive immune response. The following dynamical system describes within-host HIV infection kinetics:

$$\begin{array}{l} \dot{S}={\text{α}}_{S}-{\text{δ}}_{S}S-\text{β}SV\\ \dot{A_{P}}=(1-\text{λ})\text{τβ}SV-{\text{δ}}_{I}A_{P}-\text{κ}A_{P}E\\ \dot{A_{U}}=(1-\text{λ})(1-\text{τ})\text{β}SV-{\text{δ}}_{I}A_{U}-\text{κ}A_{U}E\\ \dot{L_{P}}=\text{λτβ}SV\\ \dot{L_{U}}=\text{λ}(1-\text{τ})\text{β}SV\\ \dot{E}={\text{α}}_{E}+\text{ω}E\frac{\left(A_{P}+A_{U}\right)}{E+E_{50}}-{\text{δ}}_{E}E\\ \dot{V}=\text{π}A_{P}-\text{γ}V-\text{β}SV \end{array}$$

The parameters used were: susceptible cell creation rate α_*S*_ = 70 cells μL^−1^ day^−1^; susceptible cell death rate δ_*S*_ = 0.2 *day*^−1^; viral infectivity β = 10^−4^μL viral RNA copies^−1^ day^−1^; probability of productively infectious virions τ =  0.05; probability of latency λ = 10^−4^; actively infected cell death rate δ_*I*_ = 0.8 day^−1^; viral burst size π =  50,000 viral RNA copies cell^−1^ day^−1^; viral clearance rate λ =  23 day^−1^; initial adaptive precursor frequency α_*E*_ = 10^−4^ cells μL^−1^ day^−1^; adaptive immune killing rate κ =  0.3 μ*L* cells^−1^ day^−1^; adaptive immune recruitment rate ω =  1.6 *day*^−1^; adaptive immune clearance rate δ_*E*_ = 0.002 day^−1^; adaptive immune 50% saturation constant [i.e., the number of infected cells required for a half-maximal cytolytic expansion rate (Reeves et al., 2017)] *E*_50_ = 250 cells μL^−1^.

The following initial conditions were used as per the recommendation of the model creator Dr. Daniel Reeves, who kindly provided us a link to his source code.^1^ We take as an initial value for the viral load *V*(0) = 0.03 viral RNA copies per μ*L*, which represents the detectable plasma viral load (pVL) limit of a typical assay when converted from the conventional 30 HIV RNA copies/mL to viral RNA copies per microliter, as required by the model. Also let *I*_0_ = *V*(0)γ/π=1.38×10^−5^; this represents a quasistatic approximation for the infected cells.

• $S(0)={\text{α}}_{S}/{\text{δ}}_{S}=70/0.2=350$
• $A_{P}(0)=I_{0}\text{τ(1}-\text{λ)}=6.89931\times 10^{-7}$
• $A_{U}(0)=I_{0}(1-\text{τ})(1-\text{λ})=1.310869\times 10^{-5}$
• $L_{P}(0)=I_{0}\text{τλ}=6.9\times 10^{-11}$
• $L_{U}(0)=I_{0}(1-\text{τ})\text{λ=1.311}\times {\text{10}}^{-9}$
• $E(0)={\text{α}}_{E}/{\text{δ}}_{E}=0.0001/0.0002=0.05$
• V(0)=30/1000=0.03

Note that *S*(0) and *E*(0) are the equilibrium values for the system in the absence of virus.

As described above, reservoir creation is assumed to be proportional to pVL over time, with the probability of a virus entering the latent pool (defined by λ above) remaining constant over time.

In our initial application of the model, where the purpose was solely to illustrate how the rate of proviral turnover during untreated infection influences proviral composition at ART initiation, we reconstructed “representative” within-host pVL dynamics using a published model that features typical HIV acute-phase pVL kinetics (Robb et al., 2016) and subsequent setpoint (Reeves et al., 2017).

Using these equations, we modeled proviral deposition into the latent pool and grouped the resulting proviruses by year of creation. In a separate step, we then allowed each group of latent proviruses to decay exponentially, at various rates (half-lives), up until the proviral sampling date. This produced a series of distributions that predict what proportion of proviruses would remain from each creation year at the time of sampling, assuming that proviruses had been eliminated from the pool at the stated rate.

### Inference of Pre-ART Proviral Half-Lives From Proviral Distributions Sampled on ART

Pre-ART proviral turnover rates *in vivo* can be inferred from age distributions of proviruses sampled on ART, because at the time of proviral sampling the majority of proviral turnover would have already occurred prior to ART. Our primary method of inferring pre-ART proviral half-life involved the application of a Poisson generalized linear model to each participant’s observed proviral distribution at time of sampling. To do this, we grouped each participant’s proviral sequences by their year of creation (integration) relative to their ART initiation date. We will refer to the bin containing proviruses from *t-1* to *t* years old as “bin *t*,” where we make the simplifying assumption that all proviruses in bin *t* are exactly *t* years old. We then applied a Poisson generalized linear model with the canonical natural logarithm link function to the binned counts, using the age of the bin *t* as the predictor. This choice can be justified as follows: Let *t*_*1/2*_ be the proviral half-life. Assuming the participant’s proviral reservoir decays at an exponential rate, we would expect that the size of bin *t* would be approximately

⌊C⁢exp⁡(-θ⁢t)⌋

where *C* represents the initial size of the age bin as if there was no decay, and θ = ln (2)/*t*_1/2_.

Next, we assume that every provirus has the same small independent probability *p* of being sampled. If so, we would then expect the number of observed proviruses from bin *t* to be binomially distributed with ⌊*C* exp (−θ*t*)⌋ trials and success probability *p*. Assuming that *p* is small, we can approximate the distribution with a Poisson distribution with parameter

λ=C⁢p⁢exp⁡(-θ⁢t)=exp⁡(D-θ⁢t)

where *D* = ln (*Cp*), or in other words,

ln⁡λ=D-θ⁢t.

The above equation is precisely the setup required for a Poisson generalized linear model with natural logarithm link function.

The above-described method infers pre-ART proviral half-lives solely from participants’ sampled proviral age distributions without incorporating any information from their clinical histories. As a sensitivity analysis, we additionally adapted the dynamical mathematical model, described earlier, to incorporate each participant’s available pre-ART pVL history to estimate proviral deposition during this period, and further extended the model to identify the pre-ART proviral half-life that best fit each participant’s observed proviral distribution.

### Statistical Analysis

Implementation of the mathematical model and calculation of within-host proviral half-lives using the Poisson generalized linear model was performed using R. All other statistical analyses were performed in GraphPad prism (version 9.0.1) using nonparametric tests.

## Results

### Estimating the Ages of Proviruses Persisting on ART

**FIGURE 1:**
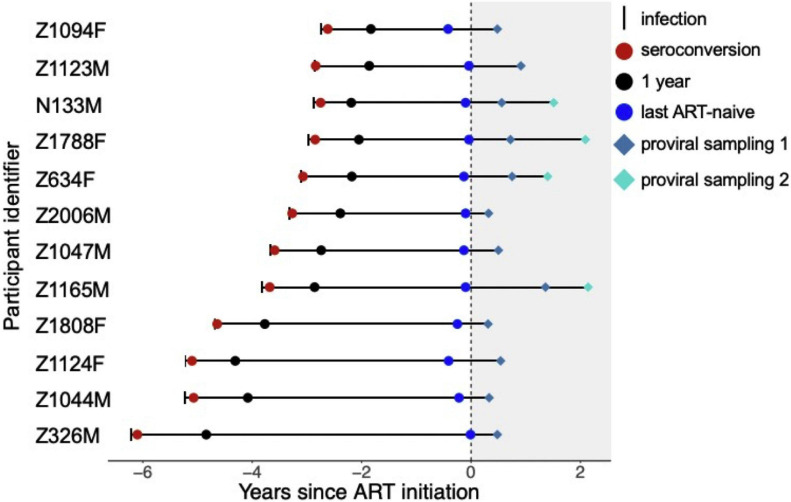
Participant sampling timeline. Infection and sampling timelines for the 12 study participants, sorted by their untreated infection duration, and using ART initiation as the reference time-point. IDs ending in “M” and “F” denote male and female participants, respectively. The short vertical line denotes the estimated date of infection, and the gray shaded area denotes ART. The three colored circles denote the dates when plasma HIV RNA *env* sequences were sampled: at seroconversion (red circle), 1 year after infection (black circle) and before ART (blue circle). The colored diamonds denote when proviral sequences were sampled on ART. Figure adapted from Brooks et al. (2020).

The study participants comprised 12 Zambian seroconverters with HIV subtype C, seven males and five females, who initiated ART within a median of 3.5 (IQR 3.0–4.8) years following infection (Figure 1). As previously described (Brooks et al., 2020), plasma HIV RNA *env* sequences were collected at three time points during untreated infection (at seroconversion, at 1 year following infection, and before ART) and proviral *env* sequences were collected a median of 6.3 (IQR 5.3–8.7) months after ART. For four participants, proviral sequences were also collected at a second time point, a median of 22 months after ART.

**FIGURE 2:**
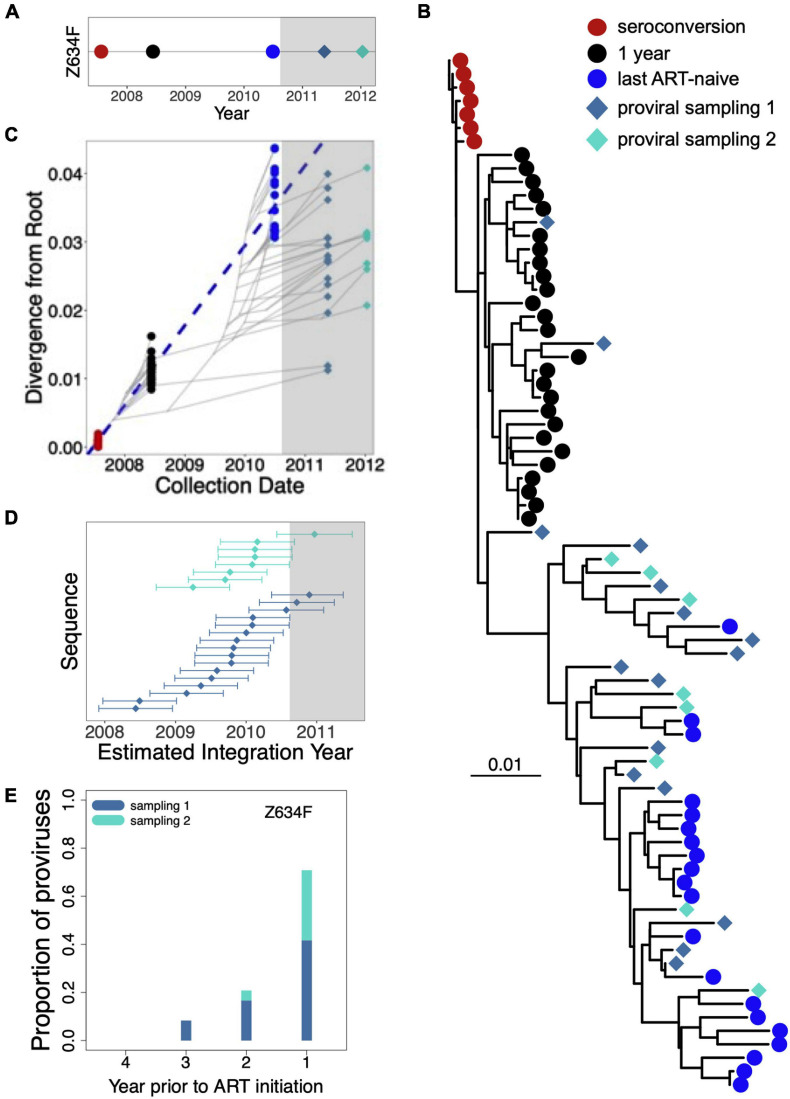
Estimating the ages of proviruses persisting on ART: Participant Z634F. **(A)** Sampling timeline for participant Z634F. Plasma HIV RNA *env* sequences were sampled at three time points between infection and ART (colored circles); proviral sequences were sampled twice on ART (colored diamonds). Gray shading denotes ART. **(B)** Within-host maximum-likelihood phylogeny inferred from intact, distinct HIV sequences, where the root represents the inferred transmitted/founder virus and the symbols at the tree tips denote the sequence type (plasma HIV RNA versus proviral) and sampling time-point. **(C)** The dashed blue line represents the linear regression relating the root-to-tip distances of the plasma HIV RNA sequences to their sampling times. The slope of the regression line represents the evolutionary rate (ER) of plasma HIV RNA *env* sequences in this participant during untreated infection (3.2 × 10^− 5^ estimated nucleotide substitutions/site/day); this line is used to convert the root-to-tip distances of proviral sequences sampled on ART to their original “creation” (i.e., integration) dates. The faint gray lines represent the underlying evolutionary relationships between sampled HIV sequences. **(D)** Point estimates and associated 95% CIs of the integration dates of sampled proviral sequences, as inferred from the regression, colored by sampling date. **(E)** Proportion of Z634F’s proviruses that dated to each year prior to ART.

The “creation” (i.e., integration) dates of each participant’s proviral sequences were estimated phylogenetically as described (Brooks et al., 2020). As these dates form the basis of our proviral half-life estimates, we explain how they are derived using participant Z634F, whose proviral pool was sampled twice on ART, as an example (Figure 2A). The majority of Z634F’s proviral sequences either intersperse with plasma HIV sequences that circulated just prior to ART, or cluster within intermediate clades between this and the preceding plasma time point, which represents viruses that circulated 1 year post-infection (Figure 2B). Two proviruses fall within the clade of plasma HIV sequences from 1 year post-infection, but none intersperse with plasma sequences collected at seroconversion. A linear model relating the root-to tip distances of pre-ART plasma HIV RNA sequences to their collection dates (Figure 2C) allows us to convert the root-to-tip distances of proviral sequences to their integration dates, along with the 95% CIs around these point estimates (Figure 2D). Grouping these proviruses by their year of integration relative to ART initiation shows that Z634F’s proviral pool predominantly (∼70%) dated to the year preceding ART (Figure 2E).

### Illustrating How Different Pre-ART Decay Rates Influence Proviral Distribution on ART

**FIGURE 3:**
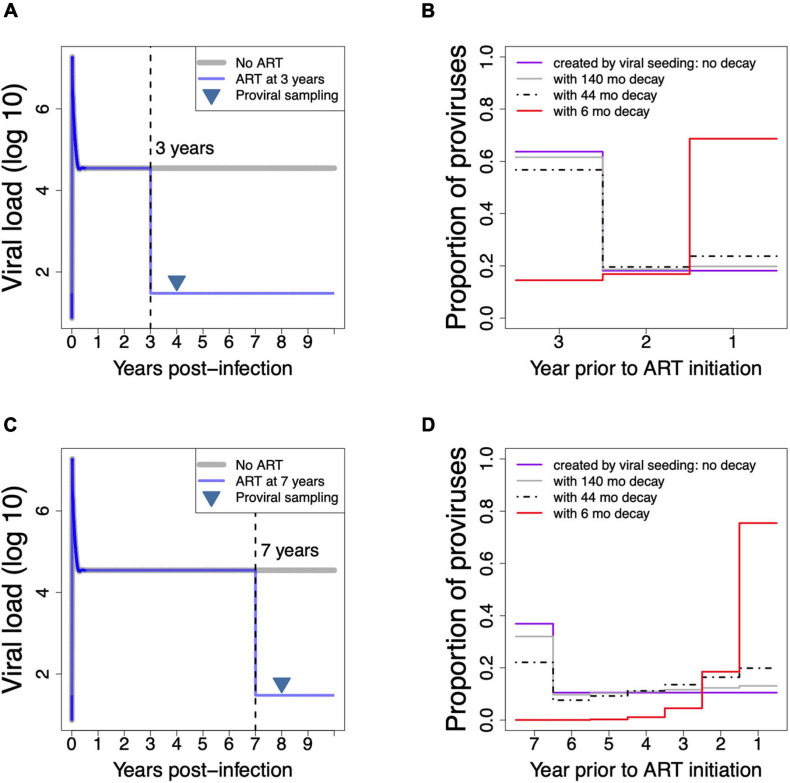
Using a mathematical model of HIV infection to demonstrate how untreated HIV infection duration and proviral decay rate shape proviral composition on ART. **(A)** Model-produced plasma viral load dynamics in a hypothetical individual who initiated ART 3 years after infection (blue line), where proviral DNA was sampled 1 year later (inverted triangle). Note that the proviral sampling time-point is illustrative only: as proviral seeding in the model ceases at ART initiation but decay proceeds at a constant rate, and because proviral compositions are depicted as *proportions* of proviruses remaining from each year of creation, model predictions would be the same regardless of when the proviral pool is sampled on ART. **(B)** Model-predicted proviral compositions on ART for the hypothetical individual shown in **A**, depicted in terms of the proportions of proviruses remaining from each year of creation, under conditions of no decay (purple line), under slow decay rates of 140 months (gray line) and 44 months (dotted black line) that represent published half-lives of proviral DNA and the replication-competent reservoir during ART, respectively (Siliciano et al., 2003; Golob et al., 2018), and under a rapid decay rate of 6 months (red line). **(C)** Same as panel **A**, but for a hypothetical individual who initiated ART 7 years after infection. **(D)** Model predicted proviral compositions on ART for the hypothetical individual shown in **C**, under various rates of decay.

We next wished to demonstrate how the duration of untreated infection, and the rate of decay of the long-lived proviral pool during this period, influence proviral age distribution on ART. To do this we employed a mathematical model of continual viral seeding into cellular HIV reservoirs followed by continual elimination, and we implemented this model for untreated infection durations that were representative of our cohort participants’ clinical histories (Figure 3). Figure 3A depicts model-generated pVL dynamics in a hypothetical individual who initiated ART 3 years after infection, and whose proviral pool was sampled 1 year thereafter. Figure 3B shows four model predictions of the individual’s proviral age distribution on ART, assuming that HIV sequences entered the reservoir during untreated infection at a rate proportional to their abundance in plasma at the time and were subsequently eliminated at a constant exponential rate. The purple line represents the total proviral pool created by viral seeding: here, the model predicts that the biggest (>60%) proportion of persisting proviruses entered the reservoir during the first year of infection (i.e., 3 years before ART initiation), because peak viral load during this period is >2 log_10_ higher than the subsequent setpoint. We also modeled what the persistent proviral pool would resemble if, after seeding, proviruses were subsequently cleared at rates comparable to those during suppressive ART. To do this we applied half-lives of 140 months [the decay rate of proviral DNA on ART (Golob et al., 2018)] and 44 months [the decay rate of the replication-competent reservoir on ART (Siliciano et al., 2003)] up until the date of sampling. This produced the predicted proviral distributions shown by the gray and black dashed lines, respectively. The differences between these distributions and the “no decay” condition are modest, due to the relatively short time from infection and ART initiation: under these relatively slow decay rates, the biggest portion of the reservoir is still predicted to constitute proviruses that integrated in the first year of infection.

By contrast, applying a relatively fast decay rate up until the date of sampling, namely a half-life of 6 months, produced a proviral distribution that is markedly enriched in sequences that integrated in the year prior to ART, though sequences from earlier in infection are still present at lower frequencies (red line). Figures 3C,D depict model-generated pVL dynamics and predicted proviral age distributions, respectively, in a hypothetical individual who initiated ART 7 years following infection, and whose proviral pool was sampled 1 year thereafter. Here, applying published “on-ART” decay rates still produce proviral distributions that are enriched in sequences that integrated in the first year of infection, though less dramatically so than the first example, because the longer untreated infection duration allows for more decay. By contrast, the fast decay rate (6-month half-life) produces a markedly skewed proviral pool where nearly 80% of sequences date to the year prior to ART, and where essentially no proviruses remain from the early years of infection. This mathematical model of HIV infection therefore clearly illustrates how faster proviral clearance rates during untreated infection skew proviral composition toward sequences seeded later in infection. Indeed, Z634F’s proviral distribution, shown in Figure 2E, more resembles that created by the faster (6 months) clearance rate than the known (far slower) rates of proviral clearance on ART.

### Inferring Proviral Half-Lives From Phylogenetically Estimated Proviral Age Distributions

**FIGURE 4:**
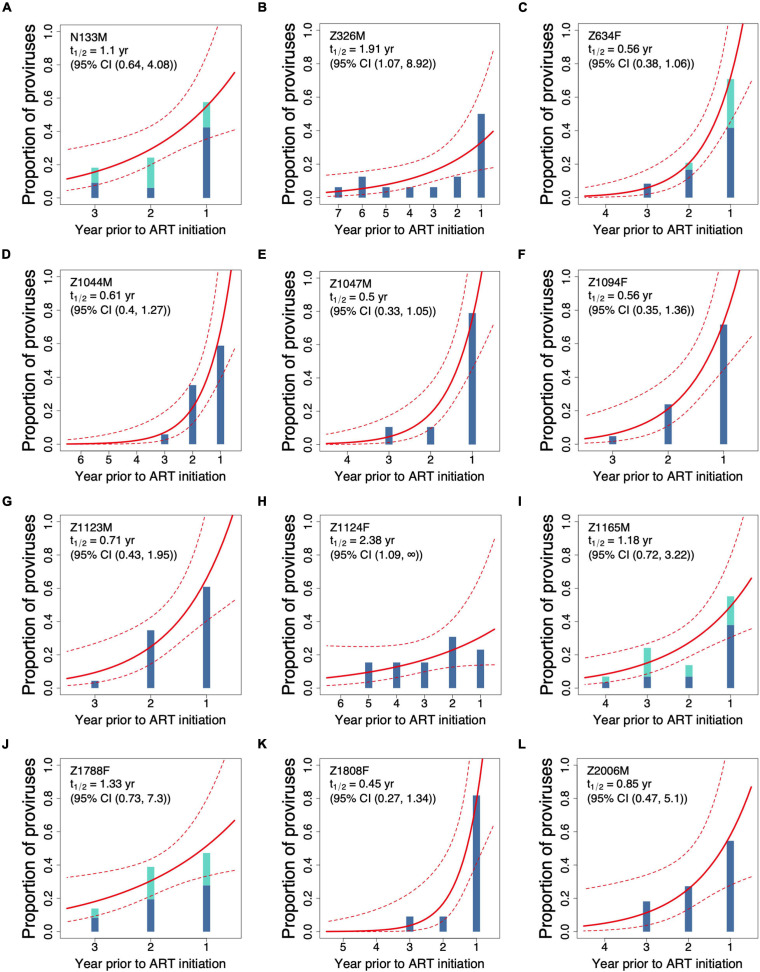
Best-fitting proviral decay rates inferred from participants’ proviral compositions on ART **(A–L)**. Each participant’s proviral distribution on ART, as determined phylogenetically *via* the procedure outlined in Figure 2, is depicted as histograms that show the proportions of proviruses remaining from each year of integration. For the four participants whose proviral pools were sampled twice on ART (N133M, Z634F, Z1165M, and Z1788F), proviral composition is shown as stacked bars. The solid and dashed red lines represent the best-fit half-life and associated 95% confidence intervals, respectively, estimated using a Poisson generalized linear model. Participants are sorted by study ID.

The phylogenetically inferred proviral age distributions for all 12 study participants, grouped by their year of creation relative to ART initiation, are shown in Figure 4. For the four participants for whom proviral DNA was sampled twice on ART, including Z634F (Figure 2), all proviral sequences were combined to maximize sampling depth. This was done because proviral decay on ART is extremely slow [half-life >10 years (Golob et al., 2018; Peluso et al., 2020)] and because proviral sampling in these individuals was performed only an average of 0.9 years apart. The data clearly illustrate the variation in participants’ proviral age distributions: while some participants’ proviral pools are highly enriched in sequences that integrated in the year preceding ART initiation, for example, Z1808F (Figure 4K) and Z1047M (Figure 4E), the proviral pools of others, for example, Z1124F (Figure 4H) feature proviruses that integrated throughout untreated infection at relatively comparable frequencies.

Proviral half-lives were inferred from participants’ proviral age distributions using a Poisson generalized linear model. This yielded proviral half-lives ranging from 0.45 years (95% CI 0.27–1.34) in participant Z1808F, for whom >80% of their proviral pool dated to the year preceding ART (Figure 4K), to 2.38 (95% CI 1.09–∞) years in Z1124F (Figure 4H), the participant with the “flattest” overall proviral age distribution. Z1124F was also the only participant for whom an upper 95% confidence bound could not be defined, indicating that we cannot reject the null hypothesis of no or extremely slow pre-ART decay in this individual. Overall, the median pre-ART proviral half-life was 0.78 (IQR 0.56–1.22) years.

**FIGURE 5:**
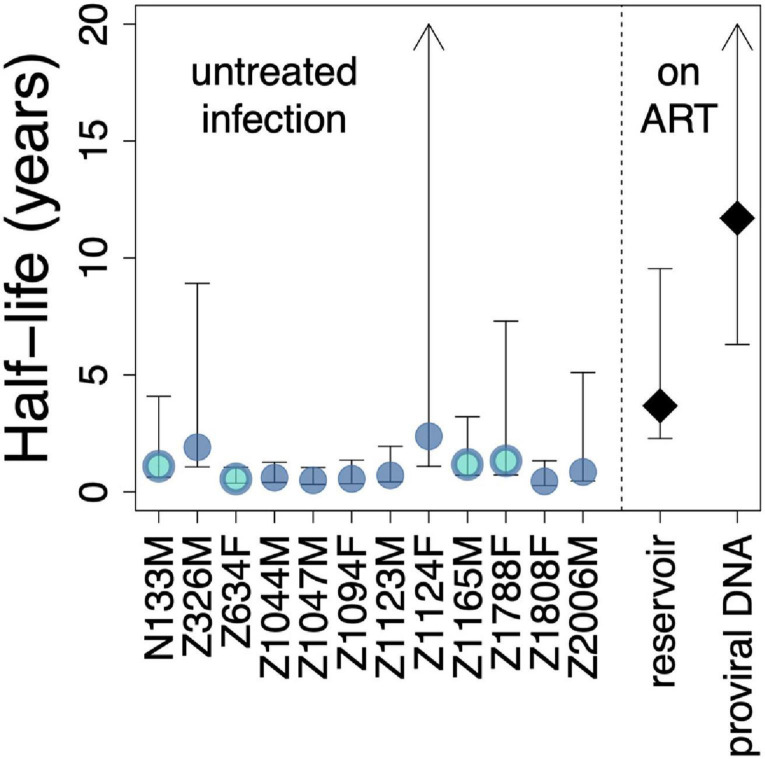
Comparison of estimated pre-ART proviral decay rates with published rates of reservoir and proviral decay on ART. Estimated pre-ART proviral half-lives and associated 95% CI are shown for the 12 participants, alongside published rates of reservoir (Siliciano et al., 2003) and proviral (Golob et al., 2018) decay on ART. Bi-colored circles represent the four participants for whom proviral sampling was performed twice on ART. Arrowheads indicate upper 95% CIs of infinity.

To compare these estimated pre-ART half-lives to published decay rates on ART, we plotted each participant’s half-life point estimate and associated 95% CI alongside historic estimates of the decay rate of the replication-competent reservoir on ART, estimated as 3.7 years (i.e., 44.2 months) with a 95% CI of 2.3–9.5 years (Siliciano et al., 2003) and the decay rate of total proviral DNA on ART, estimated as 11.7 years (i.e., 140.4 months) with a 95% CI of 6.3–240 years (Golob et al., 2018; Figure 5). Despite inter-individual variation, visualizing the data in this way reveals that 9 of the 12 participants’ estimated proviral half-lives during untreated infection are significantly lower than published overall proviral decay rate on ART, as indicated by the lack of overlap in their respective 95% CI. Moreover, 6 of 12 participants’ pre-ART half-lives are significantly lower than published decay rates of the replication competent reservoir on ART. These observations are consistent with more rapid proviral turnover in untreated compared to treated infection in *most* individuals, though it is important to note that some individuals displayed slow pre-ART turnover rates that were not significantly different than published on-ART rates.

### Pre-ART Proviral Half-Life Correlates Inversely With Plasma Viral Load Setpoint and the Rate of CD4+ T-Cell Decline

**FIGURE 6:**
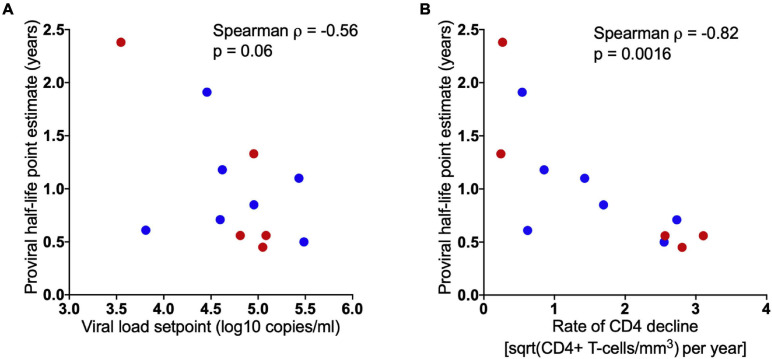
Higher pVL setpoint and rapid rate of CD4+ T-cell decline is associated with faster proviral turnover during untreated infection. Spearman’s correlation showing inverse relationship between model-estimated proviral half-lives and participant set point plasma viral load **(A)**, and rate of CD4+ T-cell decline **(B)**. Blue and red dots denote male and female participants, respectively.

Given the inter-individual variability within estimated pre-ART proviral half-lives, we sought to explore potential correlates of this decay rate. We observed no significant difference in proviral half-lives between males and females (*p* = 0.7) nor any significant relationship between proviral half-life and either the length of untreated infection (Spearman’s ρ = 0.24, *p* = 0.5) nor the time of proviral sampling following ART initiation (Spearman’s ρ = 0.4, *p* = 0.2) (data not shown). We did however observe an inverse relationship between proviral half-life and setpoint pVL, estimated as the median of all pVL measurements taken between 6 months post-infection and ART initiation (Spearman’s ρ = −0.56, *p* = 0.06; Figure 6A). Moreover, we observed a significant correlation between proviral half-life and the rate of CD4+ T-cell decline (quantified by ordinary linear regression on square-root transformed CD4+ T-cell counts measured longitudinally prior to ART, where there were a median of 13 measurements per participant) (Spearman’s ρ = −0.82, *p* = 0.0016; Figure 6B). Together, these observations suggest that higher levels of viral replication and/or faster rates of elimination of HIV infected CD4+ T-cells accelerate proviral turnover *in vivo*.

### Sensitivity and Alternative Analyses

In the following sections, we present a number of sensitivity and alternative analyses that test the robustness of our observations to various assumptions.

### Calculating Proviral Decay Rates Without Grouping Proviruses by Year of Creation

Our primary analysis grouped proviruses by year of creation prior to the initiation of ART. This was done in order to achieve a number of “bins” that was appropriate for the number of proviruses collected per participant while also recognizing that there is uncertainty around the point estimate for each provirus’ creation date. Nevertheless, to verify that our results were not unduly influenced by temporal grouping, we performed a sensitivity analysis where we applied the Poisson linear model directly to the inferred proviral dates (i.e., where we used a “bin size” of 1 day). The results of this analysis are shown in Supplementary Figures 1, 2. Overall, the within-host proviral half-life point estimates derived from this analysis were consistent with those in the primary analysis, though the 95% CIs around the estimated decay rates were generally wider. Z1124F’s estimated proviral half-life was also slower when calculated this way (8.87 versus 2.38 years in the primary analysis), though in both analyses the upper 95% CI around this estimate extended to infinity, indicating that this is not a significant difference.

### Accounting for Identical Sequences When Estimating Proviral Decay Rates

As our main objective was to infer the turnover of the persistent proviral pool pre-ART from the integration dates of individual proviruses sampled on ART, our primary analysis excluded identical sequences under the assumption that these arose *via* clonal expansion, not *via* individual integration events. We acknowledge however that by sequencing only a subgenomic HIV fragment (*env*) we cannot conclusively classify identical sequences as clonal across the whole HIV genome (Laskey et al., 2016). To account for the possibility that identical *env* sequences may represent distinct proviral genomes, we repeated all analyses including all *env* sequences collected. There were only six such “replicate” sequences across the whole dataset, among four participants (three who harbored one replicate sequence each, and one who harbored three additional replicates of one particular *env* sequence). Proviral decay rates inferred using these data are shown in Supplementary Figures 3A–D, while a summary figure showing decay rates for all participants, replicates included, is shown in Supplementary Figure 3E. Not surprisingly given the very small number of replicate sequences, results are highly consistent with the results of the primary analysis.

### Computing Proviral Decay Rates for Each Proviral Sampling Date Independently

Our cohort included four participants whose proviral pool was sampled at two time points spaced relatively shortly apart, and our primary analysis pooled these proviruses together to maximize sampling depth. In a sensitivity analysis, we estimated proviral decay rates for these two time points separately (Supplementary Figure 4). For all four participants, the 95% CI around the proviral half-life estimates derived from the first, second and combined time points overlapped one another, where all point-estimates were markedly lower than the published on-ART decay rate for proviral DNA (Golob et al., 2018). Moreover, for two of the four participants (Z634F and Z1788F), the point-estimates were nearly identical across all analyses. For the remaining two participants (N133M and Z1165F) the proviral half-life estimated from the second sampling time point was slower than that estimated by the first, though the associated 95% CI extended to infinity in both cases. For consistency with the other participants for whom proviruses were sampled only once, we recalculated the correlation between proviral half-life and pre-ART clinical parameters restricting to the proviral half-life calculated from the first sampling time point only. The inverse correlation with pVL setpoint remained (Spearman’s *R* = −0.59, *p* = 0.046; Supplementary Figure 4B), as did the correlation with CD4 decline (Spearman’s R = −0.71, *p* = 0.012; Supplementary Figure 4C).

### Incorporating Participants’ Pre-ART pVL Histories Into Proviral Half-Life Estimates

Our primary method of inferring pre-ART proviral half-lives did not incorporate any information from the participants’ clinical histories. To address this, we adapted the dynamical mathematical model (see methods and Pankau et al., 2020) to incorporate each participant’s available pre-ART pVL history, and extended the model to identify the pre-ART proviral half-life that best fit each participant’s observed proviral distribution. Note that peak viremia was not captured for the majority (10/12) of participants due to the challenges of sampling during this stage, so for these we inferred a viremia peak at 14 days after their estimated date of infection at a value that was 50 times the average of their measured pVL from 30 to 365 days post-infection. This approach was chosen because it gave peak viral load values that were consistent with those observed in acute HIV infection (van Loggerenberg et al., 2008; Mlisana et al., 2014; Dong et al., 2018). Two participants (Z1047M and Z1808F) had a pVL measurement performed during the period when they were HIV p24^*Gag*^ antigen positive yet HIV antibody negative [i.e., Fiebig stage 2 (Fiebig et al., 2003)], this measurement was used as their inferred peak. We then used a piecewise linear function to couple each participant’s inferred peak to their available longitudinal pVL data. Each participant’s pVL dynamics reconstructed in this manner are shown in Supplementary Figure 5.

We then applied the published dynamical mathematical model to predict what each participant’s proviral distribution would resemble at their time of sampling, assuming that their proviral pool had been continually seeded during untreated infection in proportion to their viral load at the time, and where this proviral pool had subsequently decayed at various rates. To do this, we allowed each group of latent proviruses created by the model to decay exponentially, under half-lives ranging from 30 to 6000 days, in increments of 30 days, up until the participants’ proviral sampling date. This yielded a series of 200 proviral distributions per participant that represented what proportion of proviruses would still remain from each creation year, assuming decay at the stated rate. For context, we also predicted reservoir composition under decay rates of 44 and 140 months [which represent the half-life of the replication-competent reservoir (Siliciano et al., 2003) and total proviral DNA (Golob et al., 2018) on ART, respectively] as well as under conditions of no decay. Although each participant’s model-generated proviral pool was allowed to decay up to their proviral sampling date, it is important to note that any date post-ART would produce the same proviral distribution. This is because, in the model, creation of new latent HIV sequences ceases after ART initiation: after this time, total reservoir *size* decreases but the overall proportion of proviruses remaining from each year of creation does not change. As a final step, we compared all of the model-generated proviral distributions to each participant’s observed proviral age distribution, and identified the decay rate (half-life) that best fit the data by maximum likelihood. We used standard theory to identify 95% confidence bounds around this decay rate estimate.

Model-predicted proviral compositions under different decay rates are shown alongside the participants’ observed proviral distributions in Supplementary Figure 6. Notably, the model-predicted proviral distributions under decay rates of 44 and 140 months (i.e., “on-ART” decay rates) fit the participants’ observed proviral distributions poorly in all cases. Instead, the proviral half-lives that best fit each participant’s observed proviral distributions were much faster than this and ranged from 0.33 years (Z1047M) to 0.99 years (Z326M), where the upper bound of the 95% CI around these best-fit estimates were, in all cases, below the lower bound of the 95% CI around the on-ART proviral DNA decay rate (Supplementary Figure 7). Though these half-life estimates assume that the reconstructed acute-phase dynamics reflect the participants’ true dynamics during this stage, and that within-host HIV replication and latency occur as parameterized in the model, these results nevertheless further support the notion that proviral turnover pre-ART is faster than on-ART.

## Discussion

The rate of proviral turnover during untreated infection is critical to HIV cure research efforts because it determines reservoir composition at ART initiation. Assuming that reservoir seeding begins shortly following infection, which is supported by studies of early ART (Jain et al., 2013; Ananworanich et al., 2015, 2016) as well as within-host phylogenetic studies that have recovered proviruses dating back to transmission or early infection (Brodin et al., 2016; Jones et al., 2018; Abrahams et al., 2019; Brooks et al., 2020; Pankau et al., 2020), if subsequent proviral turnover during untreated infection was slow, a substantial proportion of “old” sequences would continue to persist even if ART is initiated late (Figure 3). By contrast, if turnover was fast, the proviral pool would rapidly shift to younger sequences. Studying reservoir turnover during untreated infection has historically been challenging, however, as it ideally requires serial within-host sampling beginning early enough to reconstruct the transmitted/founder virus. Furthermore, even when such cohorts are available, estimating these rates solely from pre-therapy samples is imperfect because one cannot distinguish the minority of persisting proviruses from the dominant population of short-lived proviruses that are continually generated through ongoing infection. Recently however, studies that have coupled within-host HIV evolutionary reconstructions with proviruses sampled on ART, where the latter represent “true” persisting proviruses, have offered opportunities to investigate this (Brodin et al., 2016; Jones et al., 2018; Abrahams et al., 2019; Brooks et al., 2020; Pankau et al., 2020).

Here, we estimated pre-ART proviral half-lives in 12 seroconverters with HIV subtype C for whom the ages of proviruses sampled on ART had been determined phylogenetically (Brooks et al., 2020). Despite inter-individual variation, we estimated the median pre-ART proviral half-life to be 0.78 years. To our knowledge, only two studies have estimated pre-ART HIV proviral turnover using similar methods (Brodin et al., 2016; Pankau et al., 2020). Our median half-life is essentially identical to that estimated in a study of 10 individuals, most of whom were men infected with HIV subtype B (Brodin et al., 2016), though the inter-individual variability we observed is more consistent with a study of six women with HIV superinfection that reported half-life estimates ranging from 10 to 68 months (0.83–5.6 years), also using HIV *env* sequences (Pankau et al., 2020). Overall, our observation that in 75% of our study participants, the 95% CI of the pre-ART proviral half-life estimate did not overlap that of the published on-ART proviral decay rate, further supports the notion that proviral turnover is much faster during untreated compared to treated infection (Brodin et al., 2016; Abrahams et al., 2019; Pankau et al., 2020). It further suggests that this is true across the sexes, as well as across the major HIV group M subtypes.

Our observations thus corroborate the notion that ART induces a dramatic slowing of the rate of proviral turnover, thereby creating an environment that further promotes proviral persistence. Indeed, the recent study that observed marked skewing of the HIV reservoir toward sequences that integrated near the time of ART initiation concluded that “*ART alters the host environment in a way that allows the formation or stabilization of most of the long-lived latent HIV-1 reservoir*” (Abrahams et al., 2019). We propose that “stabilization” is a more appropriate term to describe this phenomenon, to avoid the misunderstanding that reservoir “formation” (i.e., creation or seeding) only begins coincident with or after ART initiation. The relatively frequent recovery of proviruses that date back to acute/early infection during ART clearly indicates that some proviruses can persist long-term during untreated infection. The term “stabilization” recognizes that reservoir seeding occurs throughout untreated infection, but that the relatively dynamic turnover during this period in most individuals means that early proviruses have a high probability of being cleared before ART is initiated. By dramatically slowing the rate of proviral decay, ART stabilizes the proviral pool in its present state, which for many individuals comprises a pool that is enriched in contemporary sequences with comparably fewer proviruses dating to earlier infection. Indeed, a mechanism for ART-driven reservoir stabilization has recently been proposed, namely that uncontrolled HIV infection skews the memory CD4+ T-cell response to a short-lived effector phenotype with lower frequencies of long-lived memory CD4+ T-cells, possibly due to dysregulated IL-7/IL7R signaling (Goonetilleke et al., 2019). As a result, during untreated infection, most proviruses are eliminated along with the short-lived effector CD4+ T-cells that harbor them, with relatively few such cells transitioning back to long-lived memory cells. Suppression of viremia on ART, however, largely restores CD4+ T-cell homeostasis, including restoration of the CD4+ T-cell transition from effector to long-lived memory T-cells, allowing proviruses within the latter to persist long-term. A related hypothesis proposes that the chronic immune activation and recurring polyclonal T-cell activation that occur during untreated infection create an environment where new provirus-containing clones dynamically replace existing ones, which are continually “washed out” as a result of their activation, differentiation, and eventual death (Grossman et al., 2020). Confirmation of the underlying mechanism may in turn yield opportunities for intervention, which may include combining ART with agents that block the CD4+ T-cell effector-to-memory transition to inhibit “stabilization” of proviruses within these cells during this critical period (Goonetilleke et al., 2019) or by therapeutically triggering sequential waves of polyclonal CD4+ T-cell activation (with concomitant enhancement of HIV protein expression) during ART, to mimic the relatively rapid cellular “washout” that occurs during untreated HIV infection (Grossman et al., 2020).

Our identification of pVL setpoint and pre-ART rate of CD4+ T-cell decline as correlates of pre-ART proviral clearance rate also extends prior understanding. To our knowledge such relationships have not previously been demonstrated in HIV, though a similar correlation with pVL setpoint was reported in a longitudinal Simian Immunodeficiency Virus (SIV) study in animals carrying a specific Major Histocompatibility Complex allele, where researchers estimated overall proviral turnover during untreated infection by comparing the frequency of a specific immune-driven viral escape mutation in RNA versus DNA during this time (Reece et al., 2012). The researchers observed very slow proviral turnover in animals who naturally controlled viremia, in whom wild-type (unescaped) proviral DNA persisted at high levels over long periods, but increasingly rapid turnover in animals with higher viral loads, in whom escape mutant proviral DNA rapidly replaced the wild-type founder virus. The correlation in the present study is not nearly as strong as that in the SIV study, because the very rapid proviral turnover in the animals with very high pVL was driven by high levels of short-lived proviral DNA generated through active SIV replication, that numerically overwhelmed the persistent SIV pool. By contrast, by sampling our proviruses during suppressive ART, we can be more confident that these represent a persisting proviral pool, whose average half-life would be expected to be orders of magnitude longer than the actively replicating pool. Our observation that both pVL setpoint and pre-ART rate of CD4+ T-cell decline correlate with pre-ART proviral clearance rate, where the latter correlation is even stronger than the former, is important because it supports the notion that, even during untreated infection, higher levels of viral replication and/or more rapid loss of CD4+ T-cells create conditions that are less favorable to proviral persistence. Indeed, the observed correlation between pre-ART proviral turnover and CD4+ decline is consistent with the notion that rapid CD4+ T-cell turnover during untreated infection is a barrier to proviral longevity during this period. Larger studies however will be required however to tease apart whether pVL and CD4+ T-cell dynamics independently correlate with pre-ART proviral clearance rate.

There are several caveats and limitations to this study, many of which are common to within-host HIV evolutionary studies. First, due to the labor-intensive nature of collecting large within-host datasets of single-genome HIV sequences over long time-scales, both the number of participants (*n* = 12) and the number of “date-able” proviruses collected per participant (median 20) are relatively modest. The latter refers to the fact that hypermutated, grossly defective and putative within-host recombinant proviruses cannot be dated phylogenetically, and were removed prior to analysis, which also means that these types of sequences are not accounted for in our pre-ART proviral half-life estimates. Our analyses are also based on inference of a single phylogeny per participant, as published in the original study (Brooks et al., 2020), but we acknowledge that this approach does not account for inherent uncertainty in within-host phylogenetic reconstruction. Moreover, there is uncertainty in our integration date estimates (e.g., see 95% CI in Figure 2D), but our methods of inferring proviral half-life incorporate information from the point estimate only. Nevertheless, our cohort size, sampling depth, and method of inferring proviral half-lives from integration date point estimates are comparable to prior studies (Brodin et al., 2016; Pankau et al., 2020).

Secondly, because we only amplified HIV *env*, we cannot distinguish intact from defective proviral genomes; in fact it is reasonable to assume that the majority of sampled proviruses harbored defects, likely large deletions, outside *env* (Sanchez et al., 1997; Ho et al., 2013; Bruner et al., 2016; Imamichi et al., 2016). Our study therefore cannot address whether, during untreated infection, the half-life of intact, replication-competent proviruses differs from that of the overall pool. We nevertheless hypothesize that this is likely to be the case, based on two observations. The first is that, on ART, intact proviruses decay more quickly than the overall proviral DNA pool (Siliciano et al., 2003; Golob et al., 2018; Pinzone et al., 2019; Gandhi et al., 2020), and it is not unreasonable that the same may be true during untreated infection, particularly if a major mechanism of elimination of such cells is *via* immune-mediated killing following activation and presentation of viral antigens. The second observation supporting the faster decay of intact versus defective proviruses during untreated infection is that the extent of skewing of replication-competent reservoirs on ART toward late infection sequences (Abrahams et al., 2019) seems to be more pronounced than that of the overall proviral pool (Brodin et al., 2016; Jones et al., 2018; Brooks et al., 2020; Pankau et al., 2020). Specifically, a median of ∼78% of replication competent reservoirs were found to date to the year preceding ART (Abrahams et al., 2019), compared to only ∼60% of overall proviruses as estimated in a 2016 study (Brodin et al., 2016). In the present dataset, a median of 58% (range 23–82%) of distinct proviruses dated to the year preceding ART. Studies that infer the integration dates of both intact and defective proviruses on ART will be required to address this.

It is also worth explicitly pointing out that the methods used to compute on-ART decay rates (which are based on longitudinal measurements of reservoir *size* on ART) differ from those used here to infer pre-ART decay rates (which leverage the age distributions of proviruses sampled at a single timepoint on ART, to infer decay rates prior to that point). The different approaches are required because it is not possible to measure the persisting proviral pool during untreated infection (because it is numerically overwhelmed by the short-lived actively replicating proviral pool, which cannot be distinguished from the persisting pool using current technologies). This distinction is worth noting because, at all stages of infection, proviral decay is likely counterbalanced to some extent by proliferation/clonal expansion in many individuals. As such, when calculating on-ART proviral half-lives from longitudinal reservoir *size* measurements during therapy, this counterbalancing will serve to underestimate the actual decay rate (i.e., if there was no proliferation, inferred on-ART decay rates would be faster than currently reported). At first it may seem that the methods used in the present study, which rely on collection of individual HIV sequences, may not be confounded by proliferation in the same way, and thus produce half-life estimates that may not be directly comparable to those estimated on ART. To some extent however, the present approaches do capture the possibility of proliferation/clonal expansion during untreated infection, albeit in a different way. This is because, at the time of sampling, we recover proviruses that have persisted in the host, regardless of mechanism (i.e., it is possible that some of the proviruses sampled may have persisted up to that point only through clonal expansion). Finally, it is also worth re-iterating that our methods (as well as those of prior studies including Brodin et al., 2016 and Pankau et al., 2020) assume that proviral decay occurs exponentially up until the sampling time, which in the present study is relatively shortly after ART-mediated pVL suppression.

## Conclusion

In conclusion, our observations reveal inter-individual variability in the rate of pre-ART proviral turnover, including one individual whose upper estimate bound is inclusive of an infinite half-life. This variation is important to keep in perspective, as it underscores the uniqueness of every individual’s HIV reservoir. In particular, although on-ART proviral pools tend to be enriched in sequences that date to advanced chronic infection, consistent with relatively rapid proviral turnover during untreated infection, older sequences are not uncommonly recovered. Furthermore, some individuals harbor proviruses from throughout their infection history at relatively equal frequencies, indicating that pre-ART proviral turnover is not rapid in all persons. Indeed, our identification that pVL setpoint and pre-ART rate of CD4+ T-cell decline correlate with pre-ART proviral clearance rate strongly supports the notion that viral and host factors influence pre-ART reservoir stability, which merits investigation in larger studies. Despite this variability, we estimate that the average half-life of persisting proviruses during untreated infection is 0.78 years, a turnover rate that is more than 15 times faster than that of proviral DNA during suppressive ART. Taken together with previous findings (Brodin et al., 2016; Abrahams et al., 2019; Pankau et al., 2020), our observations are consistent with the notion that active viral replication and rapid CD4+ T-cell depletion create an environment that is less favorable to proviral persistence, while conditions of viral suppression create a milieu that are more favorable to proviral persistence, and where ART stabilizes the proviral pool by dramatically slowing its rate of decay. Ours and previous observations further suggest that this is true across both sexes, as well as across the major HIV group M subtypes. Therapeutic strategies to inhibit this stabilizing effect or to enhance reservoir turnover during suppressive ART could therefore represent additional strategies to reduce the HIV reservoir.

## Data Availability Statement

The sequences analyzed in this study can be found in online repositories. The names of the repository/repositories and accession number(s) can be found in the article. The code for the analyses is available in the github repository (https://github.com/cfe-lab/ReservoirModelling).

## Ethics Statement

The studies involving human participants were reviewed and approved by the University Teaching Hospital Ethics Committee in Lusaka, Zambia, and additional approvals for sample and data use were granted by the Institutional Review Boards of Emory University, Simon Fraser University, and Providence Health Care/University of British Columbia. The patients/participants provided their written informed consent to participate in this study.

## Author Contributions

KB, FO, RL, CB, EH, and ZB conceived the study and analyzed the data. KB, BJ, JJ, and EH developed methods and/or contributed the data. RL implemented the mathematical models. HS contributed to data visualization. FO and ZB drafted the initial manuscript with all authors contributing to further editing. All authors approved the submitted version.

## Conflict of Interest

The authors declare that the research was conducted in the absence of any commercial or financial relationships that could be construed as a potential conflict of interest. The handling editor declared a past collaboration with the authors ZB and CB.

## Publisher’s Note

All claims expressed in this article are solely those of the authors and do not necessarily represent those of their affiliated organizations, or those of the publisher, the editors and the reviewers. Any product that may be evaluated in this article, or claim that may be made by its manufacturer, is not guaranteed or endorsed by the publisher.

## Footnotes

1
https://github.com/dbrvs/seeding/blob/master/modeling.ipynb
