# Supplementary material for: Proviral Turnover During Untreated HIV Infection Is Dynamic and Variable Between Hosts, Impacting Reservoir Composition on ART
Source: Front Microbiol. 2021 Aug 19;12:719153. doi: 10.3389/fmicb.2021.719153 (PMC8417368; doi:10.3389/fmicb.2021.719153)
Supplement: Supplementary file 1 [file Data_Sheet_1.PDF]

Figure S1

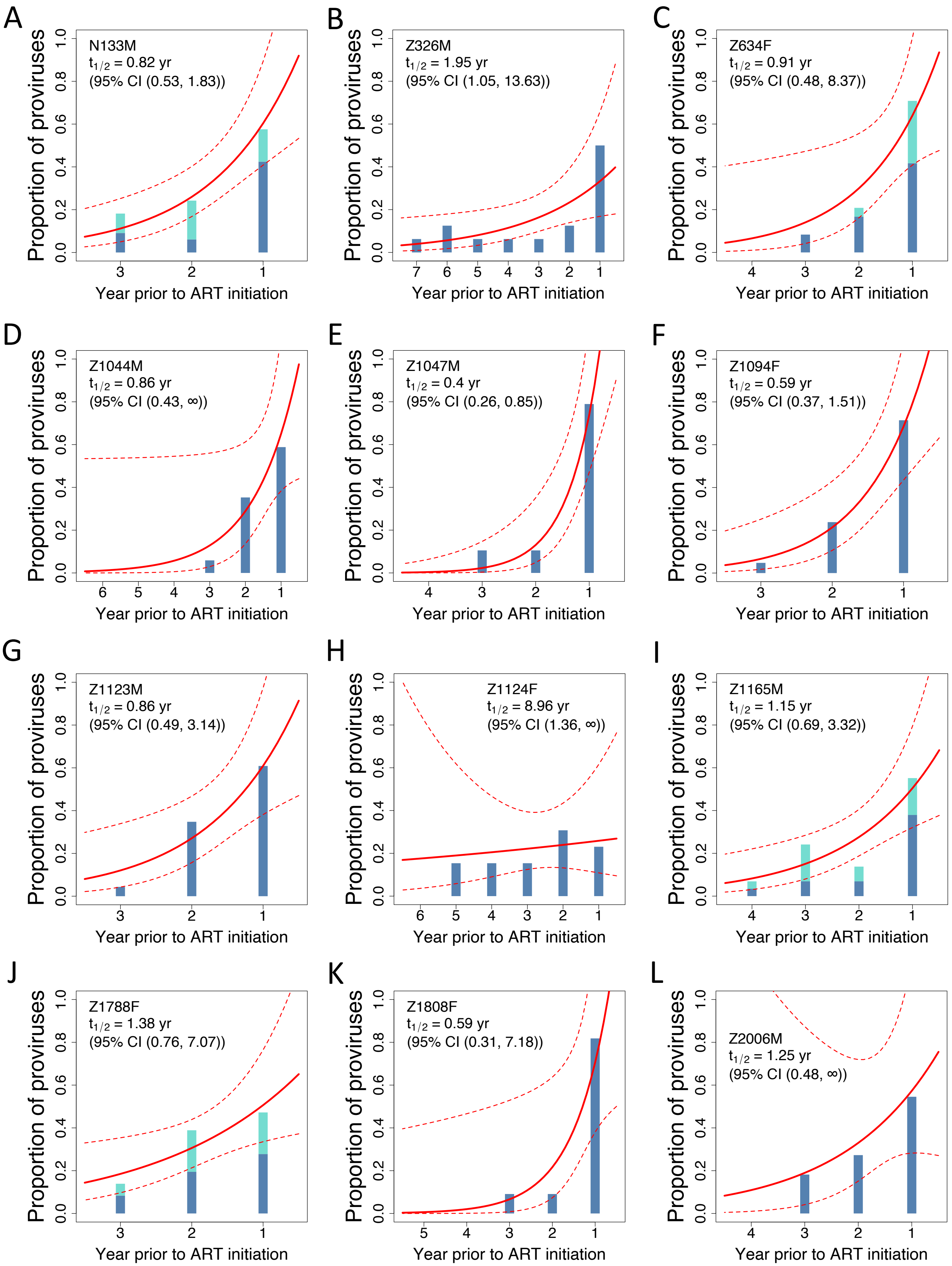
A
N133M
t1/2 = 0.82 yr
(95% CI (0.53, 1.83))
B
Z326M
t1/2 = 1.95 yr
(95% CI (1.05, 13.63))
C
Z634F
t1/2 = 0.91 yr
(95% CI (0.48, 8.37))
D
Z1044M
t1/2 = 0.86 yr
(95% CI (0.43, ∞))
E
Z1047M
t1/2 = 0.4 yr
(95% CI (0.26, 0.85))
F
Z1094F
t1/2 = 0.59 yr
(95% CI (0.37, 1.51))
G
Z1123M
t1/2 = 0.86 yr
(95% CI (0.49, 3.14))
H
Z1124F
t1/2 = 8.96 yr
(95% CI (1.36, ∞))
I
Z1165M
t1/2 = 1.15 yr
(95% CI (0.69, 3.32))
J
Z1788F
t1/2 = 1.38 yr
(95% CI (0.76, 7.07))
K
Z1808F
t1/2 = 0.59 yr
(95% CI (0.31, 7.18))
L
Z2006M
t1/2 = 1.25 yr
(95% CI (0.48, ∞))
Proportion of proviruses
Year prior to ART initiation

Figure S1 (previous page): Best-fitting proviral decay rates estimated directly from the proviral integration dates, without "binning". This analysis is analogous to that presented in Figure 4, but instead of fitting the decay rates to proviruses "binned" by year of creation prior to ART, we fit the proviral decay rates directly to the proviral integration date point estimates without "binning". Note that, for data visualization purposes, proviruses are still depicted by year of creation (where the participants whose proviruses were sampled twice on ART are shown as stacked bars), but the Poisson generalized linear model is fit directly to the individual dates. The solid and dashed red lines represent the best-fit pre-ART half-life, and associated 95% confidence interval, respectively.

Figure S2

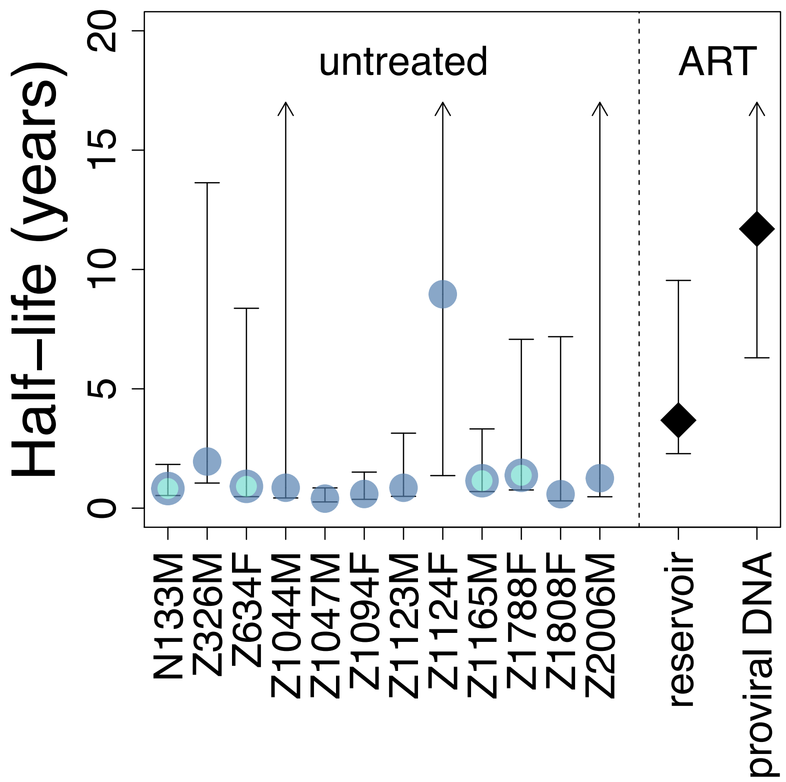

Figure S2: Comparison of estimated pre-ART proviral decay rates computed without "binning", with published rates of reservoir and proviral decay on ART. This figure is analogous to Figure 5, except the pre-ART proviral decay rates are those computed directly from the proviral point estimates without "binning" by year (*i.e.* the rates shown in Figure S1). Estimated pre-ART proviral half-lives and associated 95% CI are shown for the 12 participants, alongside published rates of reservoir (Siliciano et al., 2003) and proviral (Golob et al., 2018) decay on ART. Bi-colored circles represent the four participants for whom proviral sampling was performed twice on ART. Arrowheads indicate upper 95% CIs of infinity.

Figure S3

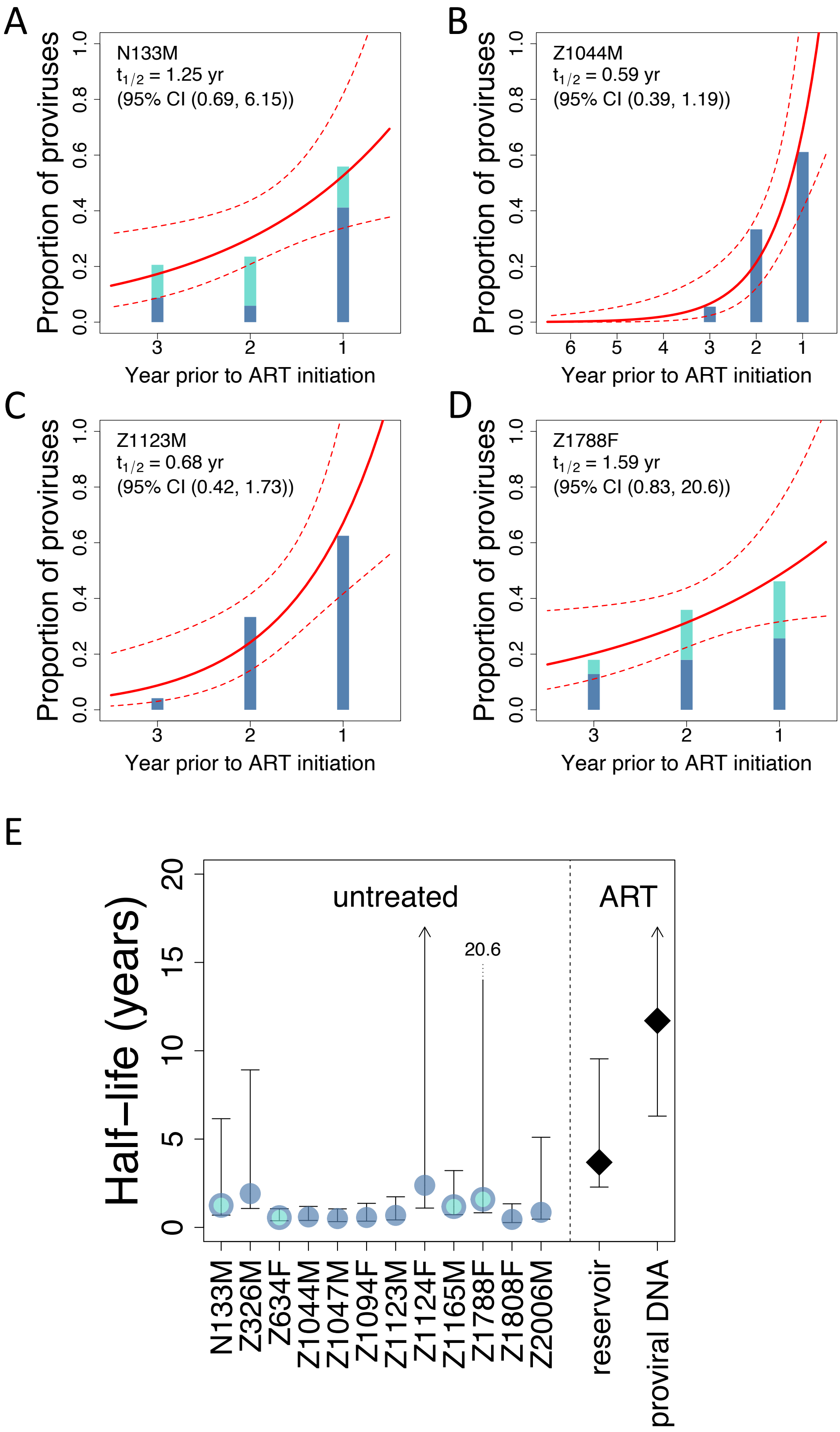

A
N133M
t1/2 = 1.25 yr
(95% CI (0.69, 6.15))
Proportion of proviruses
Year prior to ART initiation
B
Z1044M
t1/2 = 0.59 yr
(95% CI (0.39, 1.19))
Proportion of proviruses
Year prior to ART initiation
C
Z1123M
t1/2 = 0.68 yr
(95% CI (0.42, 1.73))
Proportion of proviruses
Year prior to ART initiation
D
Z1788F
t1/2 = 1.59 yr
(95% CI (0.83, 20.6))
Proportion of proviruses
Year prior to ART initiation
E
Half−life (years)
untreated
ART
20.6
N133M
Z326M
Z634F
Z1044M
Z1047M
Z1094F
Z1123M
Z1124F
Z1165M
Z1788F
Z1808F
Z2006M
reservoir
proviral DNA

Figure S3 (previous page): Best-fitting proviral decay rates inferred from all proviral sequences collected, regardless of sequence uniqueness. *Panel A-D***:** Best-fitting proviral decay rates, inferred from all proviral sequences collected regardless of uniqueness, for the four participants whose datasets featured duplicate proviral sequences. For the participants whose proviral pools were sampled twice on ART, proviral composition is shown as stacked bars. The solid and dashed red lines represent the best-fit half-life and associated 95% confidence intervals, respectively, estimated using a Poisson generalized linear model on proviruses "binned" by creation year. *Panel E*: Analogous to that presented in Figure 5, except for the four participants shown in *A-D*, we display their pre-ART proviral decay rates inferred from all their proviral sequences.

Figure S4

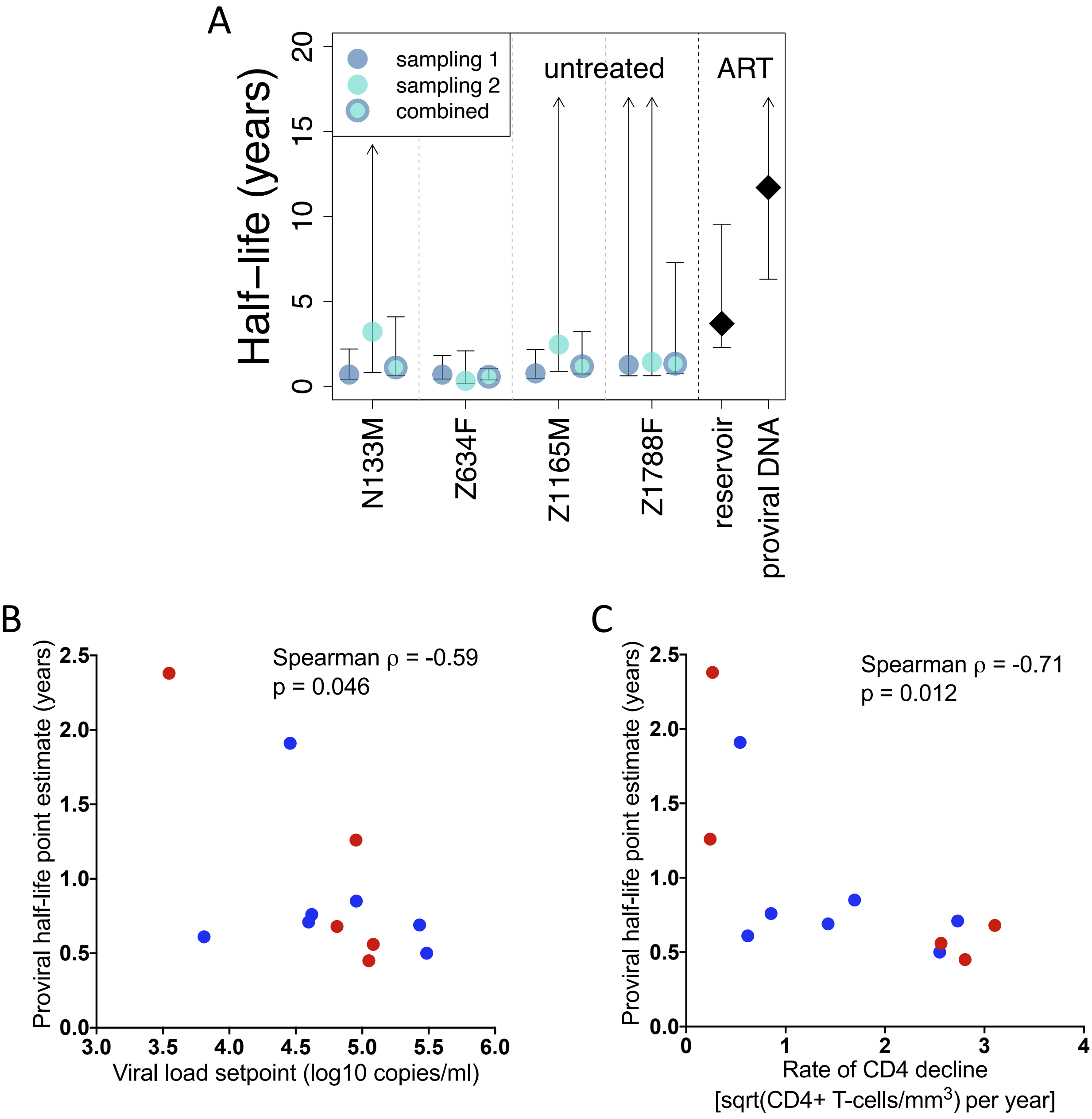

Figure S4: **Best-fitting proviral decay rates inferred from individual versus combined proviral sampling timepoints.** *Panel A:* Best-fitting proviral decay rates and associated 95% confidence intervals, inferred from individual (single-colored circles) versus combined (bi-colored circles) proviral sampling timepoints, for the four participants for whom proviral sampling was performed twice on ART. Rates were computed using a Poisson generalized linear model on proviruses "binned" by creation year. Results shown for the combined timepoint (bi-colored circles) are the same data as shown in Figure 5. Published rates of reservoir (Siliciano et al., 2003) and proviral (Golob et al., 2018) decay on ART, and associated 95% CI are shown alongside. Arrowheads indicate upper 95% CIs of infinity. *Panel B*: Analogous to the results in Figure 6A, except the correlation between plasma viral load setpoint and proviral half-life is based on the proviral half-life calculated from participants' first sampling timepoint only. Blue and red dots denote male and female participants, respectively. *Panel C*: Analogous to the results in Figure 6B, except the correlation between pre-ART rate of CD4 decline and proviral half-life is based on the proviral half-life calculated from participants' first sampling timepoint only.

Figure S5

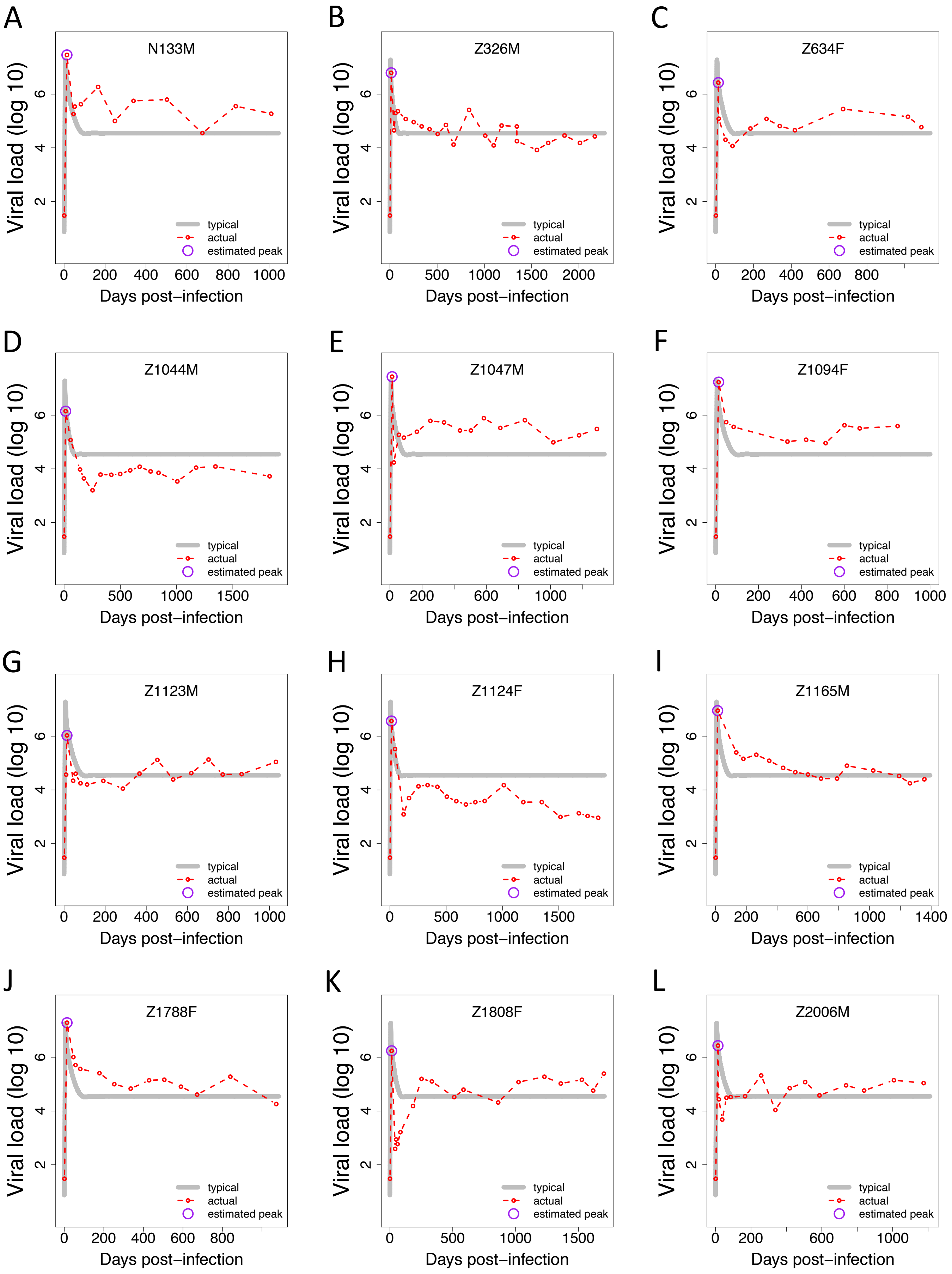
A
N133M
B
Z326M
C
Z634F
D
Z1044M
E
Z1047M
F
Z1094F
G
Z1123M
H
Z1124F
I
Z1165M
J
Z1788F
K
Z1808F
L
Z2006M
Viral load (log 10)
Days post−infection
typical
actual
estimated peak

Figure S5 (previous page): Pre-ART plasma viral load histories for all participants. The red dots represent individual viral load measurements for each participant, in terms of log_{10} HIV RNA copies/mL plasma, with dotted lines connecting these measurements. For all participants, peak viremia (shown as open purple circle) was estimated as outlined in the manuscript. For context, the grey line represents the model-produced "typical" viral load dynamic curve that was used in Figure 3. Participants are sorted by study ID.

Figure S6

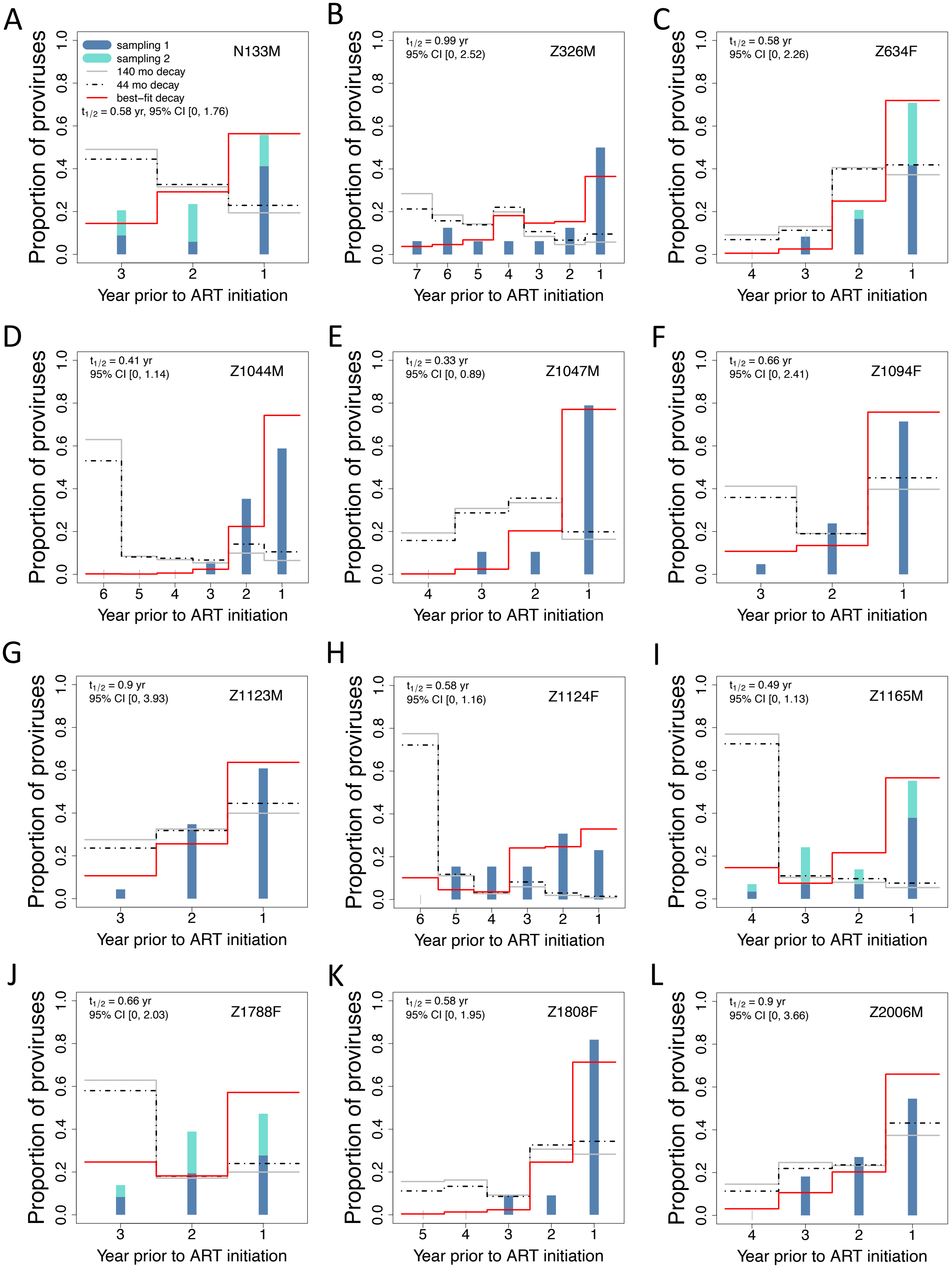

A
B
C
D
E
F
G
H
I
J
K
L
sampling 1
sampling 2
140 mo decay
44 mo decay
best–fit decay
$t_{1/2}$ = 0.58 yr, 95% CI [0, 1.76)
N133M
$t_{1/2}$ = 0.99 yr
95% CI [0, 2.52)
Z326M
$t_{1/2}$ = 0.58 yr
95% CI [0, 2.26)
Z634F
$t_{1/2}$ = 0.41 yr
95% CI [0, 1.14)
Z1044M
$t_{1/2}$ = 0.33 yr
95% CI [0, 0.89)
Z1047M
$t_{1/2}$ = 0.66 yr
95% CI [0, 2.41)
Z1094F
$t_{1/2}$ = 0.9 yr
95% CI [0, 3.93)
Z1123M
$t_{1/2}$ = 0.58 yr
95% CI [0, 1.16)
Z1124F
$t_{1/2}$ = 0.49 yr
95% CI [0, 1.13)
Z1165M
$t_{1/2}$ = 0.66 yr
95% CI [0, 2.03)
Z1788F
$t_{1/2}$ = 0.58 yr
95% CI [0, 1.95)
Z1808F
$t_{1/2}$ = 0.9 yr
95% CI [0, 3.66)
Z2006M
Proportion of proviruses
Year prior to ART initiation

Figure S6 (previous page): **Best-fitting proviral decay rates inferred from participants' proviral compositions on ART, using the dynamical mathematical model of HIV infection.** As in Figure 4, each participant's proviral distribution on ART is depicted as histograms that show the proportions of proviruses remaining from each year of integration. For the participants whose proviral pools were sampled twice on ART, proviral composition is shown as stacked bars. The different lines represent model-predicted proviral compositions on ART, depicted in terms of the proportions of proviruses that are predicted to remain from each year of infection, assuming that proviral seeding was proportional to the plasma viral load at the time, and subsequent decay proceeded at the stated rate. Model-predicted proviral distributions are shown under conditions of 140 months (grey line) and 44 months (dotted black line) decay rates, where these rates represent published half-lives of proviral DNA and the replication-competent reservoir during ART, respectively (Golob et al., 2018; Siliciano et al., 2003). The red line represents the half-life that best fit the participant's proviral distribution; this rate and its associated 95% CI is also stated as text on each panel.

Figure S7

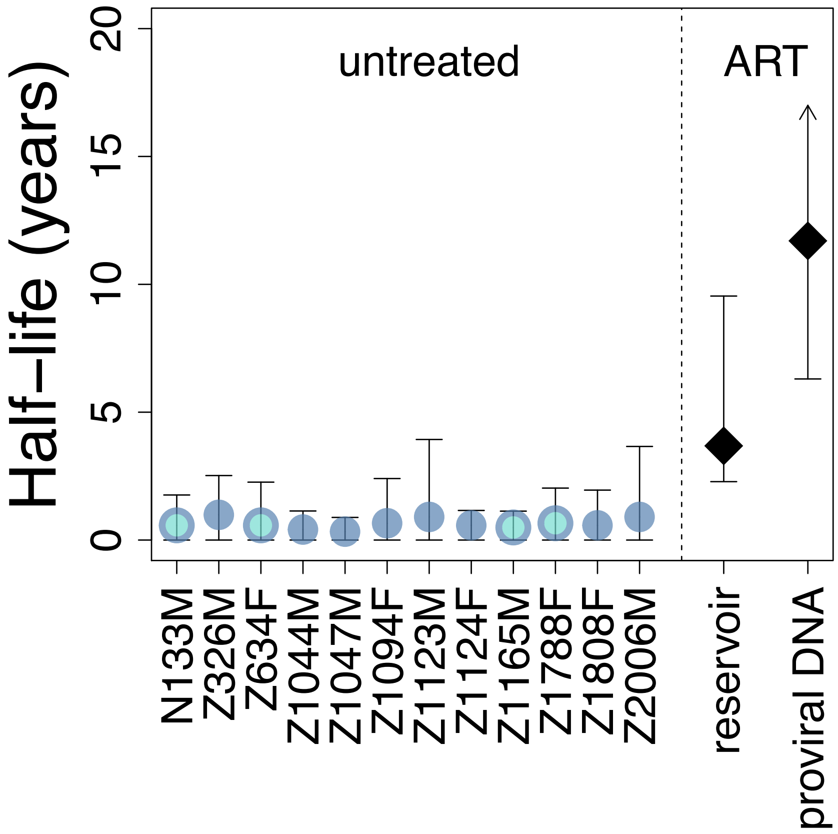

Figure S7: **Comparison of estimated pre-ART proviral decay rates, computed using the dynamical mathematical model of HIV infection, with published rates of reservoir and proviral decay on ART.** This figure is analogous to that presented in Figure 5, except the pre-ART proviral decay rates are those computed using the dynamical mathematical model of HIV infection (*i.e.* the rates shown in Figure S6). Estimated pre-ART proviral half-lives and associated 95% CI are shown for the 12 participants, alongside published rates of reservoir (Siliciano et al., 2003) and proviral (Golob et al., 2018) decay on ART. Bi-colored circles represent the four participants for whom proviral sampling was performed twice on ART. Arrowheads indicate upper 95% CIs of infinity.
